# Supplementary material for: Risk assessment based on a new decision-making approach with fermatean fuzzy sets
Source: PeerJ Comput Sci. 2025 Aug 28;11:e2990. doi: 10.7717/peerj-cs.2990 (PMC12453700; doi:10.7717/peerj-cs.2990)
Supplement: Supplemental Information 1 [file peerj-cs-11-2990-s001.docx]

| Papers | Years | Fuzzy Set Types | Methods | Application Area |
| --- | --- | --- | --- | --- |
| [43] | 2020 | FF | TOPSIS | Suitable Land for House Building Selection |
| [44] | 2021 | FF | MULTIMOORA | Electric Vehicle Charging Station Selection |
| [45] | 2021 | IVFF | TOPSIS | Solving MCDM Problem |
| [46] | 2021 | FF | TOPSIS | Aluminium Sheet Production Process Risk Assessment |
| [47] | 2021 | FF | WASPAS | Healthcare Waste Disposal Location Selection |
| [48] | 2022 | FF | CRITIC; TOPSIS | Hospital Service Performance Evaluation |
| [49] | 2022 | Hesitant IVFF | COPRAS | Desalination Technology Selection |
| [50] | 2022 | FF | CODAS | Smart City Project Selection |
| [51] | 2022 | IVFF | CRITIC; COPRAS | Sustainable Community Tourism Evaluation |
| [52] | 2022 | FF | SWARA | Reliable And Sustainable Energy Indicators Analysis |
| [53] | 2022 | FF | MEREC; MULTIMOORA | Renewable Energy Power Plant Location Identifying |
| [54] | 2022 | FF | COPRAS | Renewable Energy Source Selection |
| [55] | 2022 | FF | CRITIC; EDAS | Sustainable Third-Party Reverse Logistics Providers Selection |
| [56] | 2022 | FF | ITARA; MARCOS | Determining the Location of a Disinfection Facility for Hazardous Healthcare Waste during COVID-19 |
| [57] | 2022 | FF | CRITIC; MABAC | A Suitable Country to Develop the Construction of a High-Speed Railway Project Selection (Assessment of Investment Risks) |
| [58] | 2022 | Rough FF | DEMATEL | Taiwan’s Electronics Manufacturers Sustainable Development Evaluation |
| [59] | 2022 | IVFF | WASPAS | Selecting Most Appropriate E-Waste Recycling Partner Alternative from Sustainable Perspective |
| [60] | 2022 | FF | AHP; DEMATEL | Identify And Evaluate the Challenges in The Establishment of Human–Robot Collaboration in the Industrial Environment |
| [61] | 2022 | Cubic FF | MARCOS | Evaluation And Selecting Cold Chain Logistics Distribution Center |
| [62] | 2022 | FF | ELECTRE | Biomedical Material Selection |
| [63] | 2022 | FF | TOPSIS | Green Low-Carbon Port Evaluation |
| [64] | 2022 | FF | MEREC; CoCoSo | Evaluation of Urban Transport Planning |
| [65] | 2022 | FF | CoCoSo | Occupational Risk Evaluation in A Natural Gas Pipeline Construction Project |
| [66] | 2022 | FF | TOPSIS | Credit Risk Evaluation of Quantum Communications Listed Companies |
| [67] | 2022 | Hesitant FF | MULTIMOORA | Regional Green Development Level Evaluation |
| [68] | 2022 | FF | AHP | Supplier Selection in Industry 4.0 Transition |
| [69] | 2022 | FF | AHP | Online Teaching Quality Evaluation of Business Statistics Course Utilizing |
| [70] | 2023 | Hesitant FF | MAIRCA | Assessment of Agri-Food 4.0 Supply Chain Approaches |
| [71] | 2023 | FF | CRITIC; VIKOR | Assessment Of the Challenges to Renewable Energy Technologies Adoption in Rural Areas |
| [72] | 2023 | FF | FUCOM; MAIRCA | The Blockchain Technology Selection in The Logistics Industry |
| [73] | 2023 | FF | TOPSIS | Evaluation of Hospital Information System |
| [74] | 2023 | 2TLFF | PROMETHEE | Bank Manager Selection |
| [75] | 2022 | Hesitant FF | CoCoSo | Selection Of Blockchain Platform |
| [76] | 2023 | Trapezoidal FF | TOPSIS | Evaluating Blockchain Technology Efficiently and Flexibly |
| [77] | 2023 | 2TLFF | CODAS | Selection of Construction Companies and McDonald's Franchisees |
| [78] | 2023 | IVFF | CoCoSo | Assessing the barriers of digitally sustainable transportation system for persons with disabilities |
| [79] | 2023 | FF | CRITIC; SWARA | Digital Technology Selection Under Sustainable Transportation |
| [80] | 2023 | FF | MARCOS | Risk Evaluation for Occupational Hazards in Construction Operations |
| [81] | 2023 | FF | SWARA | Evaluation Of Risks Impeding Sustainable Mining |
| [82] | 2023 | FF | TOPSIS | Evaluation of Potential Sites to Localize Solar Farms |
| [83] | 2023 | FF | MARCOS | Warehouse Site Selection for The Automotive Industry |
| [84] | 2023 | IVFF | MEREC; MULTIMOORA | Evaluation Of Climate Change-Resilient Transportation Alternatives |
| [85] | 2023 | IVFF | AHP | Prioritization Of Supply Chain Digital Transformation Strategies |
| [86] | 2023 | IVFF | SWARA; PROMETHEE II | Ranking Bio-Medical Waste Management Organizations |
| [87] | 2024 | FF | AHP;  DEMATEL | Assessment Of Critical Success Factors İn The Supply Chain |
| [88] | 2024 | FF | AHP | Prioritizing Vulnerability Factors of Global Food Supply Chains |
| [89] | 2024 | FF | TOPSIS | Evaluate The Data Management Capability of Manufacturing Enterprises |
| [90] | 2024 | FF | ELECTRE | Ranking Of Asset Maintenance Process Key Performance İndicators |
| [91] | 2024 | FF | AHP | Assessment Of Food Supply Chain ​​Disruptions |
| [92] | 2024 | FF | SWARA; MABAC | Selection Of Healthcare Waste Recycling Technology |
| [93] | 2024 | IVFF | SWARA; EDAS | Selection of Energy Vehicle Battery Supplier |
| [94] | 2024 | FF | SWARA; TOPSIS | Assessing Performance in Meeting the Challenges of the Low-Carbon Energy Transition |
| [95] | 2024 | FF | MABAC | The Selection of New Energy Vehicle Power Battery Recycling Service Outlet |
| [96] | 2024 | IVFF | CRITIC;  PROMETHEE-II | Analysing The Barriers to Resilience Supply Chain Adoption İn The Food İndustry |
| [97] | 2024 | FF | LOPCOW;  COCOSO | Selecting Appropriate Cloud Vendors for Healthcare Center |
| [98] | 2024 | FF | DEMATEL; TOPSIS | Evaluating Sustainable Development Strategies in the Wire and Cable Industry |
| [99] | 2024 | FF | AHP; WASPAS | Occupational Hazards Analysis for Aquaculture Operations |
| [100] | 2024 | FF | MULTIMOORA | Evaluating Factors Affecting E-Scooter Selection |
| [101] | 2024 | FF | PROMETHEE | Selection of the Most Suitable Green Supplier for a Construction Company |
| [102] | 2024 | IVFF | SWARA; BWM, VIKOR | Determining Sustainable Strategies for Electronic Waste Management |
| [103] | 2024 | IVFF | LOPCOW; ARAS | Risk Assessment of R&D Projects in Industrial Robot Offline Programming Systems |
| [104] | 2024 | FF | COCOSO | Risk Priority of LNG Storage Tank Failure Modes |
| [105] | 2024 | IVFF | ARAS | Prioritizing Renewable Energy Technologies |
| [106] | 2024 | FF | COCOSO | Green Supplier Selection in Textile Industry |
| [107] | 2024 | FF | SWARA; TOPSIS | Autonomous Vehicle Parking Lot Selection |
| [108] | 2024 | IVFF | AHP | Assessing the Vulnerability of Urban Road Infrastructure to Seismic Activity |
| [109] | 2024 | FF | BWM; VIKOR | Selecting Health Care Waste Treatment Technology |
| [110] | 2024 | IVFF | SWARA; ARAS | Determination of Best Renewable Energy Sources |
| [111] | 2024 | FF | TOPSIS | Electric Vehicle Selection |
| [112] | 2025 | IVFF | WASPAS | Green Supply Chain Management |
| [113] | 2025 | FF | TOPSIS | Ranking the Strategies Based on Business Intelligence in the Context of Smart City |
| [114] | 2025 | FF | TOPSIS | Selecting a Site for a Mobile Tower Installation |
| [115] | 2025 | FF | BWM; WASPAS | Selecting 3D Printing Technology |
| [116] | 2025 | FF | WASPAS | Evaluating Logistics Sector Sustainability Indicators |
| Current Study | 2025 | IVFF | AHP; TOPSIS | Assessment of Plastic Injection Moulding Machine Risks |

**Note:** Fermatean Fuzzy (FF); Interval Valued Fermatean Fuzzy (IVFF); Technique for Order Performance by Similarity to Ideal Solution (TOPSIS); Multi-Objective Optimization on the basis of Ratio Analysis (MULTIMOORA); Weighted Aggregated Sum Product Assessment (WASPAS); Criteria Importance Through Intercriteria Correlation (CRITIC); Complex Proportional Assessment (COPRAS); Combinative Distance-based Assessment (CODAS); Step-wise Weight Assessment Ratio Analysis (SWARA); Method based on the Removal Effects of Criteria (MEREC); Evaluation based on Distance from Average Solution (EDAS); Inter-criteria Trade-off Analysis Ratio Assessment (ITARA); Measurement of Alternatives and Ranking according to COmpromise Solution (MARCOS); Multi-Attributive Border Approximation area Comparison (MABAC); Decision Making Trial and Evaluation Laboratory (DEMATEL); Analytic Hierarchy Process (AHP); Elimination and Choice Expressing Reality(ELECTRE); Combined Compromise Solution (CoCoSo); Multi-Attributive Ideal-Real Comparative Analysis (MAIRCA); VIšekriterijumsko KOmpromisno Rangiranje (VIKOR); Full Consistency Method(FUCOM); Preference Ranking Organization Method for Enrichment Evaluation (PROMETHEE); Logarithmic percentage change-driven objective weighting (LOPCOW); Best-Worst Method (BWM); Additive Ratio Assessment(ARAS);  2-tuple linguistic Fermatean fuzzy (2TLFF).
