# Supplementary material for: Risk assessment based on a new decision-making approach with fermatean fuzzy sets
Source: PeerJ Comput Sci. 2025 Aug 28;11:e2990. doi: 10.7717/peerj-cs.2990 (PMC12453700; doi:10.7717/peerj-cs.2990)
Supplement: Supplemental Information 11 [file peerj-cs-11-2990-s011.docx]

| n | 1 | 2 | 3 | 4 | 5 | 6 | 7 | 8 | 9 | 10 | 11 | 12 | 13 | 14 |
| --- | --- | --- | --- | --- | --- | --- | --- | --- | --- | --- | --- | --- | --- | --- |
| RI | 0 | 0 | 0.58 | 0.90 | 1.12 | 1.24 | 1.32 | 1.41 | 1.45 | 1.49 | 1.51 | 1.56 | 1.57 | 1.58 |
