# Supplementary material for: Risk assessment based on a new decision-making approach with fermatean fuzzy sets
Source: PeerJ Comput Sci. 2025 Aug 28;11:e2990. doi: 10.7717/peerj-cs.2990 (PMC12453700; doi:10.7717/peerj-cs.2990)
Supplement: Supplemental Information 12 [file peerj-cs-11-2990-s012.docx]

| DM Weights | SDMG1 |  | P1 | P2 | P3 | P4 | P5 | P6 | P7 | CR |
| --- | --- | --- | --- | --- | --- | --- | --- | --- | --- | --- |
| 0.3641 | DM1 | P1 | EI | **SLI** | SLI | SLI | EI | EI | SMI | 0,005 |
|  |  | P2 | SMI | EI | SMI | SMI | VHI | HI | VHI |  |
|  |  | P3 | SMI | SLI | EI | SMI | EI | EI | SMI |  |
|  |  | P4 | SMI | SLI | SLI | EI | EI | EI | SMI |  |
|  |  | P5 | EI | VLI | EI | EI | EI | EI | SMI |  |
|  |  | P6 | EI | LI | EI | EI | EI | EI | HI |  |
|  |  | P7 | SLI | VLI | SLI | SLI | SLI | LI | EI |  |
| 0.2718 | DM2 | P1 | EI | **LI** | SLI | CHI | SMI | HI | VHI | 0,097 |
|  |  | P2 | HI | EI | SMI | CHI | VHI | CHI | CHI |  |
|  |  | P3 | SMI | SLI | EI | CHI | HI | VHI | CHI |  |
|  |  | P4 | CLI | CLI | CLI | EI | VLI | LI | SLI |  |
|  |  | P5 | SLI | VLI | LI | VHI | EI | SMI | HI |  |
|  |  | P6 | LI | CLI | VLI | HI | SLI | EI | SMI |  |
|  |  | P7 | VLI | CLI | CLI | SMI | LI | SLI | EI |  |
| 0.3641 | DM3 | P1 | EI | **LI** | SMI | SLI | LI | LI | SLI | 0,077 |
|  |  | P2 | HI | EI | CHI | SMI | SMI | SMI | HI |  |
|  |  | P3 | SLI | CLI | EI | LI | VLI | VLI | LI |  |
|  |  | P4 | SMI | SLI | HI | EI | SMI | EI | HI |  |
|  |  | P5 | HI | SLI | VHI | SLI | EI | SLI | SMI |  |
|  |  | P6 | HI | SLI | VHI | EI | SMI | EI | SMI |  |
|  |  | P7 | SMI | LI | HI | LI | SLI | SLI | EI |  |
