# Supplementary material for: Risk assessment based on a new decision-making approach with fermatean fuzzy sets
Source: PeerJ Comput Sci. 2025 Aug 28;11:e2990. doi: 10.7717/peerj-cs.2990 (PMC12453700; doi:10.7717/peerj-cs.2990)
Supplement: Supplemental Information 13 [file peerj-cs-11-2990-s013.docx]

| DM Weights | SDGM5 |  | H1 | H2 | H3 | H4 | H5 | H6 | H7 | H8 | H9 | CR |
| --- | --- | --- | --- | --- | --- | --- | --- | --- | --- | --- | --- | --- |
| 0.2808 | DM1 | H1 | EI | **SLI** | VHI | CHI | CHI | CHI | HI | SMI | LI | 0,096 |
|  |  | H2 | SMI | EI | CHI | CHI | CHI | CHI | VHI | HI | SLI |  |
|  |  | H3 | VLI | CLI | EI | SMI | EI | SMI | SLI | LI | CLI |  |
|  |  | H4 | CLI | CLI | SLI | EI | SLI | EI | LI | CLI | CLI |  |
|  |  | H5 | CLI | CLI | EI | SMI | EI | SMI | SLI | VLI | CLI |  |
|  |  | H6 | CLI | CLI | SLI | EI | SLI | EI | VLI | CLI | CLI |  |
|  |  | H7 | LI | VLI | SMI | HI | SMI | VHI | EI | SLI | CLI |  |
|  |  | H8 | SLI | LI | HI | CHI | VHI | CHI | SMI | EI | VLI |  |
|  |  | H9 | HI | SMI | CHI | CHI | CHI | CHI | CHI | VHI | EI |  |
| 0.2808 | DM2 | H1 | EI | **SLI** | VHI | CHI | CHI | SMI | HI | HI | SLI | 0,08 |
|  |  | H2 | SMI | EI | CHI | CHI | CHI | HI | VHI | VHI | EI |  |
|  |  | H3 | VLI | CLI | EI | HI | SMI | LI | SLI | SLI | CLI |  |
|  |  | H4 | CLI | CLI | LI | EI | SLI | VLI | LI | LI | CLI |  |
|  |  | H5 | CLI | CLI | SLI | SMI | EI | VLI | LI | LI | CLI |  |
|  |  | H6 | SLI | LI | HI | VHI | VHI | EI | SMI | SMI | LI |  |
|  |  | H7 | LI | VLI | SMI | HI | HI | SLI | EI | EI | VLI |  |
|  |  | H8 | LI | VLI | SMI | HI | HI | SLI | EI | EI | VLI |  |
|  |  | H9 | SMI | EI | CHI | CHI | CHI | HI | VHI | VHI | EI |  |
| 0.2808 | DM3 | H1 | EI | **LI** | EI | EI | HI | SLI | LI | LI | LI | 0,094 |
|  |  | H2 | HI | EI | VHI | HI | HI | SMI | EI | SMI | EI |  |
|  |  | H3 | EI | VLI | EI | LI | SLI | LI | LI | LI | LI |  |
|  |  | H4 | EI | LI | HI | EI | EI | EI | LI | LI | SLI |  |
|  |  | H5 | LI | LI | SMI | EI | EI | LI | LI | LI | SLI |  |
|  |  | H6 | SMI | SLI | HI | EI | HI | EI | EI | EI | EI |  |
|  |  | H7 | HI | EI | HI | HI | HI | EI | EI | SMI | SMI |  |
|  |  | H8 | HI | SLI | HI | HI | HI | EI | SLI | EI | SMI |  |
|  |  | H9 | HI | EI | HI | SMI | SMI | EI | SLI | SLI | EI |  |
| 0.1575 | DM7 | H1 | EI | **LI** | HI | CHI | CHI | VHI | SMI | SLI | VLI | 0,099 |
|  |  | H2 | HI | EI | CHI | CHI | CHI | CHI | VHI | SMI | SLI |  |
|  |  | H3 | LI | CLI | EI | SMI | SMI | EI | SLI | VLI | CLI |  |
|  |  | H4 | CLI | CLI | SLI | EI | EI | EI | VLI | CLI | CLI |  |
|  |  | H5 | CLI | CLI | SLI | EI | EI | SLI | VLI | CLI | CLI |  |
|  |  | H6 | VLI | CLI | EI | EI | SMI | EI | SLI | CLI | CLI |  |
|  |  | H7 | SLI | VLI | SMI | VHI | VHI | SMI | EI | LI | CLI |  |
|  |  | H8 | SMI | SLI | VHI | CHI | CHI | CHI | HI | EI | LI |  |
|  |  | H9 | VHI | SMI | CHI | CHI | CHI | CHI | CHI | HI | EI |  |
