# Supplementary material for: Risk assessment based on a new decision-making approach with fermatean fuzzy sets
Source: PeerJ Comput Sci. 2025 Aug 28;11:e2990. doi: 10.7717/peerj-cs.2990 (PMC12453700; doi:10.7717/peerj-cs.2990)
Supplement: Supplemental Information 14 [file peerj-cs-11-2990-s014.docx]

| DM Weights | SDMG2 |  | H1 | H2 | H3 | H4 | H5 | H6 | H7 | H8 | H9 | CR |
| --- | --- | --- | --- | --- | --- | --- | --- | --- | --- | --- | --- | --- |
| 0.1778 | DM1 | H1 | EI | SMI | HI | CHI | VHI | LI | HI | LI | SLI | 0,087 |
|  |  | H2 | SLI | EI | SMI | VHI | HI | VLI | SMI | VLI | LI |  |
|  |  | H3 | LI | SLI | EI | HI | SMI | CLI | EI | CLI | VLI |  |
|  |  | H4 | CLI | VLI | LI | EI | SLI | CLI | LI | CLI | CLI |  |
|  |  | H5 | VLI | LI | SLI | SMI | EI | CLI | SLI | CLI | CLI |  |
|  |  | H6 | HI | VHI | CHI | CHI | CHI | EI | CHI | EI | SMI |  |
|  |  | H7 | LI | SLI | EI | HI | SMI | CLI | EI | CLI | CLI |  |
|  |  | H8 | HI | VHI | CHI | CHI | CHI | EI | CHI | EI | SMI |  |
|  |  | H9 | SMI | HI | VHI | CHI | CHI | SLI | CHI | SLI | EI |  |
| 0.1336 | DM4 | H1 | EI | SMI | CHI | CHI | VHI | SLI | HI | LI | SLI | 0,095 |
|  |  | H2 | SLI | EI | VHI | CHI | HI | LI | SMI | VLI | CLI |  |
|  |  | H3 | CLI | VLI | EI | EI | SLI | CLI | LI | CLI | CLI |  |
|  |  | H4 | CLI | CLI | EI | EI | SLI | CLI | SLI | CLI | CLI |  |
|  |  | H5 | VLI | LI | SMI | SMI | EI | CLI | SLI | CLI | CLI |  |
|  |  | H6 | SMI | HI | CHI | CHI | CHI | EI | VHI | SLI | SLI |  |
|  |  | H7 | LI | SLI | HI | SMI | SMI | VLI | EI | CLI | CLI |  |
|  |  | H8 | HI | VHI | CHI | CHI | CHI | SMI | CHI | EI | EI |  |
|  |  | H9 | SMI | CHI | CHI | CHI | CHI | SMI | CHI | EI | EI |  |
| 0.1778 | DM2 | H1 | EI | VHI | EI | CHI | CHI | VLI | LI | SLI | EI | 0,098 |
|  |  | H2 | VLI | EI | SLI | HI | SMI | CLI | CLI | CLI | SLI |  |
|  |  | H3 | EI | SMI | EI | VHI | HI | CLI | EI | EI | EI |  |
|  |  | H4 | CLI | LI | VLI | EI | EI | CLI | CLI | CLI | VLI |  |
|  |  | H5 | CLI | SLI | LI | EI | EI | CLI | CLI | CLI | LI |  |
|  |  | H6 | VHI | CHI | CHI | CHI | CHI | EI | SMI | HI | VHI |  |
|  |  | H7 | HI | CHI | EI | CHI | CHI | SLI | EI | SMI | HI |  |
|  |  | H8 | SMI | CHI | EI | CHI | CHI | LI | SLI | EI | SMI |  |
|  |  | H9 | EI | SMI | EI | VHI | HI | VLI | LI | SLI | EI |  |
| 0.1778 | DM3 | H1 | EI | VHI | HI | HI | VHI | SMI | SMI | HI | VHI | 0,088 |
|  |  | H2 | VLI | EI | LI | SLI | LI | VLI | LI | EI | LI |  |
|  |  | H3 | LI | HI | EI | SMI | SMI | SLI | SMI | HI | HI |  |
|  |  | H4 | LI | SMI | SLI | EI | SLI | LI | SLI | EI | LI |  |
|  |  | H5 | VLI | HI | SLI | SMI | EI | SLI | EI | SMI | EI |  |
|  |  | H6 | SLI | VHI | SMI | HI | SMI | EI | VHI | HI | HI |  |
|  |  | H7 | SLI | HI | SLI | SMI | EI | VLI | EI | SMI | EI |  |
|  |  | H8 | LI | EI | LI | EI | SLI | LI | SLI | EI | EI |  |
|  |  | H9 | VLI | HI | LI | HI | EI | LI | EI | EI | EI |  |
| 0.0997 | DM5 | H1 | EI | VHI | CHI | CHI | CHI | HI | HI | SMI | EI | 0,096 |
|  |  | H2 | VLI | EI | HI | EI | SMI | SLI | SLI | LI | VLI |  |
|  |  | H3 | CLI | LI | EI | EI | SLI | VLI | VLI | CLI | CLI |  |
|  |  | H4 | CLI | EI | EI | EI | LI | CLI | CLI | CLI | CLI |  |
|  |  | H5 | CLI | SLI | SMI | HI | EI | LI | LI | VLI | CLI |  |
|  |  | H6 | LI | SMI | VHI | CHI | HI | EI | EI | LI | CHI |  |
|  |  | H7 | LI | SMI | VHI | CHI | HI | EI | EI | LI | CHI |  |
|  |  | H8 | SLI | HI | CHI | CHI | VHI | HI | HI | EI | LI |  |
|  |  | H9 | EI | VHI | CHI | CHI | CHI | CLI | CLI | HI | EI |  |
| 0.0997 | DM6 | H1 | EI | HI | CHI | CHI | CHI | VHI | VHI | SMI | EI | 0.086 |
|  |  | H2 | LI | EI | VHI | CHI | HI | SMI | SMI | SLI | LI |  |
|  |  | H3 | CLI | VLI | EI | SMI | SLI | LI | LI | CLI | CLI |  |
|  |  | H4 | CLI | CLI | SLI | EI | LI | VLI | VLI | CLI | CLI |  |
|  |  | H5 | CLI | LI | SMI | HI | EI | SLI | SLI | VLI | CLI |  |
|  |  | H6 | VLI | VLI | HI | VHI | SMI | EI | EI | LI | VLI |  |
|  |  | H7 | VLI | SLI | HI | VHI | SMI | EI | EI | LI | VLI |  |
|  |  | H8 | SLI | SMI | CHI | CHI | VHI | HI | HI | EI | SLI |  |
|  |  | H9 | EI | HI | CHI | CHI | CHI | VHI | VHI | SMI | EI |  |
| 0.1336 | DM7 | H1 | EI | SMI | CHI | CHI | CHI | SMI | VHI | EI | LI | 0.098 |
|  |  | H2 | SLI | EI | VHI | VHI | HI | EI | SMI | SLI | CLI |  |
|  |  | H3 | CLI | VLI | EI | EI | SLI | CLI | LI | CLI | CLI |  |
|  |  | H4 | CLI | VLI | EI | EI | SLI | CLI | LI | CLI | CLI |  |
|  |  | H5 | CLI | LI | SMI | SMI | EI | VLI | SLI | CLI | CLI |  |
|  |  | H6 | SLI | EI | CHI | CHI | VHI | EI | HI | SLI | CLI |  |
|  |  | H7 | VLI | SLI | HI | HI | SMI | LI | EI | CLI | CLI |  |
|  |  | H8 | EI | SMI | CHI | CHI | CHI | SMI | CHI | EI | SLI |  |
|  |  | H9 | HI | CHI | CHI | CHI | CHI | CHI | CHI | SMI | EI |  |
