# Supplementary material for: Risk assessment based on a new decision-making approach with fermatean fuzzy sets
Source: PeerJ Comput Sci. 2025 Aug 28;11:e2990. doi: 10.7717/peerj-cs.2990 (PMC12453700; doi:10.7717/peerj-cs.2990)
Supplement: Supplemental Information 15 [file peerj-cs-11-2990-s015.docx]

| DM Weights | SDMG2 |  | H1 | H2 | H3 | H4 | H5 | H6 | H7 | H8 | H9 | CR |
| --- | --- | --- | --- | --- | --- | --- | --- | --- | --- | --- | --- | --- |
| 0.1778 | DM1 | H1 | EI | CLI | CLI | SLI | VLI | LI | CLI | LI | EI | 0,074 |
|  |  | H2 | CHI | EI | EI | CHI | HI | VHI | EI | VHI | CHI |  |
|  |  | H3 | CHI | EI | EI | CHI | VHI | CHI | EI | CHI | CHI |  |
|  |  | H4 | SMI | CLI | CLI | EI | LI | SLI | CLI | SLI | SMI |  |
|  |  | H5 | VHI | LI | VLI | HI | EI | SMI | LI | SMI | VHI |  |
|  |  | H6 | HI | VLI | CLI | CLI | SMI | EI | VLI | EI | HI |  |
|  |  | H7 | CHI | EI | EI | CHI | HI | VHI | EI | VHI | CHI |  |
|  |  | H8 | HI | VLI | CLI | SMI | SLI | EI | VLI | EI | HI |  |
|  |  | H9 | EI | CLI | CLI | SLI | VLI | LI | CLI | LI | EI |  |
| 0.1336 | DM4 | H1 | EI | CLI | CLI | SLI | CLI | LI | VLI | LI | EI | 0,083 |
|  |  | H2 | CHI | EI | SLI | CHI | SMI | VHI | HI | VHI | CHI |  |
|  |  | H3 | CHI | SMI | EI | CHI | HI | CHI | VHI | CHI | CHI |  |
|  |  | H4 | SMI | CLI | CLI | EI | VLI | SLI | LI | SLI | EI |  |
|  |  | H5 | CHI | SLI | LI | VHI | EI | HI | SMI | HI | CHI |  |
|  |  | H6 | HI | VLI | CLI | SMI | LI | EI | SLI | EI | HI |  |
|  |  | H7 | VHI | LI | VLI | HI | SLI | SMI | EI | SMI | VHI |  |
|  |  | H8 | HI | VLI | CLI | SMI | LI | EI | SLI | EI | HI |  |
|  |  | H9 | EI | CLI | CLI | EI | CLI | LI | VLI | LI | EI |  |
| 0.1778 | DM2 | H1 | EI | CLI | CLI | EI | CLI | CLI | CLI | SLI | EI | 0,098 |
|  |  | H2 | CHI | EI | SMI | CHI | HI | SLI | LI | VHI | CHI |  |
|  |  | H3 | CHI | SLI | EI | CHI | SMI | LI | VLI | HI | VHI |  |
|  |  | H4 | EI | CLI | CLI | EI | VLI | CLI | CLI | SLI | EI |  |
|  |  | H5 | CHI | LI | SLI | VHI | EI | VLI | CLI | SMI | HI |  |
|  |  | H6 | CHI | SMI | HI | CHI | VHI | EI | SLI | CHI | CHI |  |
|  |  | H7 | CHI | HI | VHI | CHI | CHI | SMI | EI | CHI | CHI |  |
|  |  | H8 | SMI | VLI | LI | SMI | SLI | CLI | CLI | EI | SMI |  |
|  |  | H9 | EI | CLI | VLI | LI | LI | CLI | CLI | SLI | EI |  |
| 0.1778 | DM3 | H1 | EI | VLI | VLI | SLI | VLI | LI | LI | SLI | SLI | 0,086 |
|  |  | H2 | VHI | EI | EI | VHI | HI | SMI | VHI | VHI | VHI |  |
|  |  | H3 | VHI | EI | EI | VHI | HI | HI | VHI | VHI | CHI |  |
|  |  | H4 | SMI | VLI | VLI | EI | LI | SLI | SLI | SLI | SLI |  |
|  |  | H5 | VHI | LI | LI | HI | EI | EI | SMI | HI | HI |  |
|  |  | H6 | HI | SLI | LI | SMI | EI | EI | HI | HI | SMI |  |
|  |  | H7 | HI | VLI | VLI | SMI | SLI | LI | EI | SMI | EI |  |
|  |  | H8 | SMI | VLI | VLI | SMI | LI | LI | SLI | EI | SLI |  |
|  |  | H9 | SMI | VLI | CLI | SMI | LI | SLI | EI | SMI | EI |  |
| 0.0997 | DM5 | H1 | EI | CLI | CLI | EI | EI | VLI | CLI | SLI | EI | 0,098 |
|  |  | H2 | CHI | EI | SMI | CHI | CHI | VHI | HI | CHI | CHI |  |
|  |  | H3 | CHI | SLI | EI | CHI | CHI | HI | SMI | CHI | VHI |  |
|  |  | H4 | EI | CLI | CLI | EI | EI | CLI | CLI | LI | SLI |  |
|  |  | H5 | EI | CLI | CLI | EI | EI | CLI | CLI | SLI | EI |  |
|  |  | H6 | VHI | VLI | LI | CHI | CHI | EI | SLI | HI | SMI |  |
|  |  | H7 | CHI | LI | CLI | CHI | CHI | SMI | EI | VHI | HI |  |
|  |  | H8 | SMI | CLI | CLI | HI | SMI | LI | VLI | EI | SLI |  |
|  |  | H9 | EI | CLI | VLI | SMI | EI | SLI | LI | SMI | EI |  |
| 0.0997 | DM6 | H1 | EI | CLI | CLI | HI | HI | LI | VLI | SLI | SMI | 0.098 |
|  |  | H2 | CHI | EI | EI | CHI | CHI | SMI | SMI | CHI | CHI |  |
|  |  | H3 | CHI | EI | EI | CHI | CHI | HI | SMI | VHI | CHI |  |
|  |  | H4 | LI | CLI | CLI | EI | EI | CLI | CLI | VLI | SLI |  |
|  |  | H5 | LI | CLI | CLI | EI | EI | CLI | CLI | VLI | SLI |  |
|  |  | H6 | HI | SLI | LI | CHI | CHI | EI | SLI | SMI | CHI |  |
|  |  | H7 | VHI | SLI | SLI | CHI | CHI | SMI | EI | HI | VHI |  |
|  |  | H8 | SMI | CLI | VLI | VHI | VHI | SLI | LI | EI | HI |  |
|  |  | H9 | SLI | CLI | CLI | SMI | SMI | CLI | VLI | LI | EI |  |
| 0.1336 | DM7 | H1 | EI | CLI | CLI | EI | EI | CLI | LI | VLI | SLI | 0.087 |
|  |  | H2 | CHI | EI | SLI | CHI | CHI | SMI | VHI | HI | CHI |  |
|  |  | H3 | CHI | SMI | EI | CHI | CHI | HI | CHI | VHI | CHI |  |
|  |  | H4 | EI | CLI | CLI | EI | EI | CLI | LI | VLI | SLI |  |
|  |  | H5 | EI | CLI | CLI | EI | EI | CLI | LI | VLI | SLI |  |
|  |  | H6 | CHI | SLI | LI | CHI | CHI | EI | HI | SMI | VHI |  |
|  |  | H7 | HI | VLI | CLI | HI | HI | LI | EI | SLI | SMI |  |
|  |  | H8 | VHI | LI | VLI | VHI | VHI | SLI | SMI | EI | HI |  |
|  |  | H9 | SMI | CLI | CLI | SMI | SMI | VLI | SLI | LI | EI |  |
