# Supplementary material for: Risk assessment based on a new decision-making approach with fermatean fuzzy sets
Source: PeerJ Comput Sci. 2025 Aug 28;11:e2990. doi: 10.7717/peerj-cs.2990 (PMC12453700; doi:10.7717/peerj-cs.2990)
Supplement: Supplemental Information 16 [file peerj-cs-11-2990-s016.docx]

| DM Weights | SDMG3 |  | H1 | H2 | H3 | H4 | H5 | H6 | H7 | H8 | H9 | CR |
| --- | --- | --- | --- | --- | --- | --- | --- | --- | --- | --- | --- | --- |
| 0.2461 | DM1 | H1 | EI | CHI | HI | VHI | SMI | SLI | CHI | LI | LI | 0,099 |
|  |  | H2 | CLI | EI | SLI | SLI | CLI | CLI | SLI | CLI | CLI |  |
|  |  | H3 | LI | SMI | EI | SMI | SLI | VLI | SMI | CLI | CLI |  |
|  |  | H4 | VLI | SMI | SLI | EI | LI | CLI | SMI | CLI | CLI |  |
|  |  | H5 | SLI | CHI | SMI | HI | EI | LI | VHI | VLI | VLI |  |
|  |  | H6 | SMI | CHI | VHI | CHI | HI | EI | CHI | SLI | SLI |  |
|  |  | H7 | CLI | SMI | SLI | SLI | VLI | CLI | EI | CLI | CLI |  |
|  |  | H8 | HI | CHI | CHI | CHI | VHI | SMI | CHI | EI | EI |  |
|  |  | H9 | HI | CHI | CHI | CHI | VHI | SMI | CHI | EI | EI |  |
| 0.2461 | DM2 | H1 | EI | EI | VHI | CHI | EI | SLI | SMI | VLI | LI | 0,096 |
|  |  | H2 | EI | EI | SMI | HI | EI | EI | EI | VLI | VLI |  |
|  |  | H3 | VLI | SLI | EI | SMI | CLI | CLI | SLI | CLI | CLI |  |
|  |  | H4 | CLI | LI | SLI | EI | CLI | VLI | SLI | CLI | CLI |  |
|  |  | H5 | EI | EI | CHI | CHI | EI | SLI | SMI | VLI | LI |  |
|  |  | H6 | SMI | EI | CHI | VHI | SMI | EI | HI | LI | SLI |  |
|  |  | H7 | SLI | EI | SMI | SMI | SLI | LI | EI | CLI | CLI |  |
|  |  | H8 | VHI | VHI | CHI | CHI | VHI | HI | CHI | EI | SMI |  |
|  |  | H9 | HI | VHI | CHI | CHI | HI | SMI | CHI | SLI | EI |  |
| 0.1849 | DM5 | H1 | EI | HI | VHI | CHI | CHI | SLI | SMI | LI | LI | 0,099 |
|  |  | H2 | LI | EI | SMI | HI | HI | VLI | SLI | CLI | CLI |  |
|  |  | H3 | VLI | SLI | EI | EI | EI | CLI | LI | CLI | CLI |  |
|  |  | H4 | CLI | LI | EI | EI | EI | CLI | CLI | CLI | CLI |  |
|  |  | H5 | CLI | LI | EI | EI | EI | CLI | CLI | CLI | CLI |  |
|  |  | H6 | SMI | VHI | CHI | CHI | CHI | EI | HI | SLI | SLI |  |
|  |  | H7 | SLI | SMI | HI | CHI | CHI | LI | EI | CLI | CLI |  |
|  |  | H8 | HI | CHI | CHI | CHI | CHI | SMI | CHI | EI | EI |  |
|  |  | H9 | HI | CHI | CHI | CHI | CHI | SMI | CHI | EI | EI |  |
| 0.1849 | DM6 | H1 | EI | SLI | SMI | HI | HI | VLI | LI | CLI | CLI | 0,099 |
|  |  | H2 | SMI | EI | HI | VHI | VHI | LI | SLI | VLI | VLI |  |
|  |  | H3 | SLI | LI | EI | SMI | SMI | CLI | CLI | CLI | CLI |  |
|  |  | H4 | LI | VLI | SLI | EI | EI | CLI | CLI | CLI | CLI |  |
|  |  | H5 | LI | VLI | SLI | EI | EI | CLI | CLI | CLI | CLI |  |
|  |  | H6 | VHI | HI | CHI | CHI | CHI | EI | SMI | SLI | SLI |  |
|  |  | H7 | HI | SMI | CHI | CHI | CHI | SLI | EI | LI | LI |  |
|  |  | H8 | CHI | VHI | CHI | CHI | CHI | SMI | HI | EI | EI |  |
|  |  | H9 | CHI | VHI | CHI | CHI | CHI | SMI | HI | EI | EI |  |
| 0.1380 | DM7 | H1 | EI | LI | SLI | EI | EI | CLI | VLI | CLI | CLI | 0,034 |
|  |  | H2 | HI | EI | SMI | HI | HI | LI | SLI | LI | LI |  |
|  |  | H3 | SMI | SLI | EI | SMI | SMI | VLI | LI | VLI | VLI |  |
|  |  | H4 | EI | LI | SLI | EI | EI | CLI | VLI | CLI | CLI |  |
|  |  | H5 | EI | LI | SLI | EI | EI | CLI | VLI | CLI | CLI |  |
|  |  | H6 | CHI | HI | VHI | CHI | CHI | EI | SMI | EI | EI |  |
|  |  | H7 | VHI | SMI | HI | VHI | VHI | SLI | EI | SLI | SLI |  |
|  |  | H8 | CHI | HI | VHI | CHI | CHI | EI | SMI | EI | EI |  |
|  |  | H9 | CHI | HI | VHI | CHI | CHI | EI | SMI | EI | EI |  |
