# Supplementary material for: Risk assessment based on a new decision-making approach with fermatean fuzzy sets
Source: PeerJ Comput Sci. 2025 Aug 28;11:e2990. doi: 10.7717/peerj-cs.2990 (PMC12453700; doi:10.7717/peerj-cs.2990)
Supplement: Supplemental Information 17 [file peerj-cs-11-2990-s017.docx]

| DM Weights | SDGM4 |  | H1 | H2 | H3 | H4 | H5 | H6 | H7 | H8 | H9 | CR |
| --- | --- | --- | --- | --- | --- | --- | --- | --- | --- | --- | --- | --- |
| 0.2135 | DM1 | H1 | EI | VLI | SMI | CHI | SMI | SLI | HI | LI | CLI | 0,095 |
|  |  | H2 | VHI | EI | CHI | CHI | CHI | SMI | CHI | SMI | SLI |  |
|  |  | H3 | SLI | CLI | EI | SMI | EI | LI | EI | VLI | CLI |  |
|  |  | H4 | CLI | CLI | SLI | EI | SLI | CLI | SLI | CLI | CLI |  |
|  |  | H5 | SLI | CLI | EI | SMI | EI | CLI | EI | CLI | CLI |  |
|  |  | H6 | SMI | SLI | HI | CHI | CHI | EI | CHI | SLI | VLI |  |
|  |  | H7 | LI | CLI | EI | SMI | EI | CLI | EI | CLI | CLI |  |
|  |  | H8 | HI | SLI | VHI | CHI | CHI | SMI | CHI | EI | LI |  |
|  |  | H9 | CHI | SMI | CHI | CHI | CHI | VHI | CHI | HI | EI |  |
| 0.2135 | DM2 | H1 | EI | HI | SMI | CHI | CHI | VHI | VHI | VHI | EI | 0,035 |
|  |  | H2 | LI | EI | SLI | HI | HI | SMI | SMI | SMI | LI |  |
|  |  | H3 | SLI | SMI | EI | VHI | VHI | HI | HI | HI | SLI |  |
|  |  | H4 | CLI | LI | VLI | EI | EI | SLI | SLI | SLI | CLI |  |
|  |  | H5 | CLI | LI | VLI | EI | EI | SLI | SLI | SLI | CLI |  |
|  |  | H6 | VLI | SLI | LI | SMI | SMI | EI | EI | EI | CLI |  |
|  |  | H7 | VLI | SLI | LI | SMI | SMI | EI | EI | EI | CLI |  |
|  |  | H8 | VLI | SLI | LI | SMI | SMI | EI | EI | EI | CLI |  |
|  |  | H9 | EI | HI | SMI | CHI | CHI | CHI | CHI | CHI | EI |  |
| 0.2135 | DM3 | H1 | EI | VLI | SLI | EI | SLI | SLI | VLI | VLI | CLI | 0,094 |
|  |  | H2 | VHI | EI | HI | SMI | HI | SMI | SLI | VLI | LI |  |
|  |  | H3 | SMI | LI | EI | SMI | SMI | SLI | LI | VLI | LI |  |
|  |  | H4 | EI | SLI | SLI | EI | SMI | LI | LI | VLI | LI |  |
|  |  | H5 | SMI | LI | SLI | SLI | EI | SLI | LI | VLI | VLI |  |
|  |  | H6 | SMI | SLI | SMI | HI | SMI | EI | SLI | VLI | LI |  |
|  |  | H7 | VHI | SMI | HI | HI | HI | SMI | EI | SLI | SLI |  |
|  |  | H8 | VHI | VHI | VHI | VHI | VHI | VHI | SMI | EI | EI |  |
|  |  | H9 | CHI | HI | HI | HI | VHI | HI | SMI | EI | EI |  |
| 0.1198 | DM5 | H1 | EI | EI | EI | CHI | CHI | VHI | CHI | SMI | SLI | 0,099 |
|  |  | H2 | EI | EI | SMI | CHI | CHI | VHI | VHI | HI | LI |  |
|  |  | H3 | EI | SLI | EI | CHI | CHI | HI | VHI | SMI | VLI |  |
|  |  | H4 | CLI | CLI | CLI | EI | EI | LI | SLI | VLI | CLI |  |
|  |  | H5 | CLI | CLI | CLI | EI | EI | LI | SLI | VLI | CLI |  |
|  |  | H6 | VLI | VLI | LI | HI | HI | EI | SMI | SLI | CLI |  |
|  |  | H7 | CLI | VLI | VLI | SMI | SMI | SLI | EI | LI | CLI |  |
|  |  | H8 | SLI | LI | SLI | VHI | VHI | SMI | HI | EI | CLI |  |
|  |  | H9 | SMI | HI | VHI | CHI | CHI | CHI | CHI | CHI | EI |  |
| 0.1198 | DM6 | H1 | EI | EI | SMI | CHI | CHI | HI | VHI | HI | SLI | 0,059 |
|  |  | H2 | EI | EI | SMI | CHI | CHI | HI | VHI | HI | SLI |  |
|  |  | H3 | SLI | SLI | EI | VHI | VHI | SMI | HI | SMI | LI |  |
|  |  | H4 | CLI | CLI | VLI | EI | EI | LI | SLI | LI | CLI |  |
|  |  | H5 | CLI | CLI | VLI | EI | EI | LI | SLI | LI | CLI |  |
|  |  | H6 | LI | LI | SLI | HI | HI | EI | SMI | EI | CLI |  |
|  |  | H7 | VLI | VLI | LI | SMI | SMI | SLI | EI | SLI | CLI |  |
|  |  | H8 | LI | LI | SLI | HI | HI | EI | SMI | EI | CLI |  |
|  |  | H9 | SMI | SMI | HI | CHI | CHI | CHI | CHI | CHI | EI |  |
| 0.1198 | DM7 | H1 | EI | VLI | SMI | HI | HI | EI | SMI | EI | SMI | 0.094 |
|  |  | H2 | VHI | EI | VHI | CHI | CHI | HI | CHI | SMI | EI |  |
|  |  | H3 | SLI | VLI | EI | SMI | SMI | SLI | EI | LI | CLI |  |
|  |  | H4 | LI | CLI | SLI | EI | EI | LI | SLI | VLI | CLI |  |
|  |  | H5 | LI | CLI | SLI | EI | EI | LI | SLI | VLI | CLI |  |
|  |  | H6 | EI | LI | SMI | HI | HI | EI | HI | SLI | SLI |  |
|  |  | H7 | SLI | CLI | EI | SMI | SMI | LI | EI | CLI | CLI |  |
|  |  | H8 | EI | SLI | HI | VHI | VHI | SMI | CHI | EI | SLI |  |
|  |  | H9 | SLI | EI | CHI | CHI | CHI | SMI | CHI | SMI | EI |  |
