# Supplementary material for: Risk assessment based on a new decision-making approach with fermatean fuzzy sets
Source: PeerJ Comput Sci. 2025 Aug 28;11:e2990. doi: 10.7717/peerj-cs.2990 (PMC12453700; doi:10.7717/peerj-cs.2990)
Supplement: Supplemental Information 18 [file peerj-cs-11-2990-s018.docx]

| DM Weights | SDMG4 |  | H1 | H2 | H3 | H4 | H5 | H6 | H7 | H8 | H9 | CR |
| --- | --- | --- | --- | --- | --- | --- | --- | --- | --- | --- | --- | --- |
| 0.2052 | DM1 | H1 | EI | EI | SLI | VLI | CLI | VLI | CLI | LI | EI | 0,098 |
|  |  | H2 | EI | EI | SLI | CLI | CLI | VLI | CLI | LI | EI |  |
|  |  | H3 | SMI | SMI | EI | VLI | CLI | LI | CLI | SLI | HI |  |
|  |  | H4 | VHI | CHI | VHI | EI | SLI | SMI | LI | HI | CHI |  |
|  |  | H5 | CHI | CHI | CHI | SMI | EI | HI | SLI | HI | CHI |  |
|  |  | H6 | VHI | VHI | HI | SLI | LI | EI | CLI | SMI | CHI |  |
|  |  | H7 | CHI | CHI | CHI | HI | SMI | CHI | EI | CHI | CHI |  |
|  |  | H8 | HI | HI | SMI | LI | LI | SLI | CLI | EI | CHI |  |
|  |  | H9 | EI | EI | LI | CLI | CLI | CLI | CLI | CLI | EI |  |
| 0.2052 | DM2 | H1 | EI | LI | SMI | HI | HI | VLI | VLI | EI | VHI | 0,084 |
|  |  | H2 | HI | EI | VHI | CHI | CHI | SLI | SLI | SMI | CHI |  |
|  |  | H3 | SLI | VLI | EI | SMI | SMI | CLI | CLI | LI | HI |  |
|  |  | H4 | LI | CLI | SLI | EI | EI | CLI | CLI | VLI | SMI |  |
|  |  | H5 | LI | CLI | SLI | EI | EI | CLI | CLI | VLI | SMI |  |
|  |  | H6 | VHI | SMI | CHI | CHI | CHI | EI | EI | HI | CHI |  |
|  |  | H7 | VHI | SMI | CHI | CHI | CHI | EI | EI | HI | CHI |  |
|  |  | H8 | EI | SLI | HI | VHI | VHI | LI | LI | EI | CHI |  |
|  |  | H9 | VLI | CLI | LI | SLI | SLI | CLI | CLI | CLI | EI |  |
| 0.2052 | DM3 | H1 | EI | VHI | EI | HI | SMI | SLI | VHI | VHI | HI | 0,094 |
|  |  | H2 | VLI | EI | LI | LI | LI | VLI | EI | SMI | SLI |  |
|  |  | H3 | EI | HI | EI | HI | SMI | EI | VHI | CHI | VHI |  |
|  |  | H4 | LI | HI | LI | EI | LI | VHI | EI | HI | HI |  |
|  |  | H5 | SLI | HI | SLI | HI | EI | EI | HI | VHI | VHI |  |
|  |  | H6 | SMI | VHI | EI | VLI | EI | EI | VHI | VHI | HI |  |
|  |  | H7 | VLI | EI | VLI | EI | LI | VLI | EI | HI | EI |  |
|  |  | H8 | VLI | SLI | CLI | LI | VLI | VLI | LI | EI | SLI |  |
|  |  | H9 | LI | SMI | VLI | LI | VLI | LI | EI | SMI | EI |  |
| 0.1151 | DM5 | H1 | EI | SMI | EI | VLI | LI | SLI | CLI | SLI | EI | 0,075 |
|  |  | H2 | SLI | EI | SLI | CLI | VLI | LI | CLI | LI | SLI |  |
|  |  | H3 | EI | SMI | EI | VLI | LI | SLI | CLI | SLI | EI |  |
|  |  | H4 | VHI | CHI | CHI | EI | SMI | HI | EI | HI | CHI |  |
|  |  | H5 | HI | VHI | HI | SLI | EI | SMI | EI | SMI | SMI |  |
|  |  | H6 | SMI | HI | SMI | LI | SLI | EI | EI | EI | CHI |  |
|  |  | H7 | CHI | CHI | CHI | EI | EI | EI | EI | CHI | CHI |  |
|  |  | H8 | SMI | HI | SMI | LI | SLI | EI | CLI | EI | VHI |  |
|  |  | H9 | SLI | SMI | SLI | CLI | SLI | CLI | CLI | VLI | EI |  |
| 0.1151 | DM6 | H1 | EI | HI | EI | VLI | CLI | LI | CLI | SLI | SMI | 0,093 |
|  |  | H2 | LI | EI | LI | CLI | CLI | VLI | CLI | VLI | SLI |  |
|  |  | H3 | EI | HI | EI | VLI | CLI | LI | CLI | SLI | SMI |  |
|  |  | H4 | VHI | CHI | VHI | EI | SLI | SMI | LI | EI | VHI |  |
|  |  | H5 | CHI | CHI | CHI | SMI | EI | HI | SLI | EI | CHI |  |
|  |  | H6 | HI | VHI | HI | SLI | LI | EI | VLI | EI | VHI |  |
|  |  | H7 | CHI | CHI | CHI | HI | SMI | VHI | EI | SMI | CHI |  |
|  |  | H8 | SMI | VHI | SMI | EI | EI | EI | SLI | EI | CHI |  |
|  |  | H9 | SLI | SMI | SLI | VLI | CLI | VLI | CLI | CLI | EI |  |
| 0.1542 | DM7 | H1 | EI | EI | SLI | CLI | VLI | LI | CLI | LI | EI | 0.062 |
|  |  | H2 | EI | EI | SLI | CLI | VLI | LI | CLI | LI | EI |  |
|  |  | H3 | SMI | SMI | EI | VLI | LI | SLI | VLI | SLI | SMI |  |
|  |  | H4 | CHI | CHI | VHI | EI | SMI | HI | SLI | HI | CHI |  |
|  |  | H5 | VHI | VHI | HI | SLI | EI | SMI | LI | SMI | VHI |  |
|  |  | H6 | HI | HI | SMI | LI | SLI | EI | VLI | EI | VHI |  |
|  |  | H7 | CHI | CHI | VHI | SMI | HI | VHI | EI | CHI | CHI |  |
|  |  | H8 | HI | HI | SMI | LI | SLI | EI | CLI | EI | HI |  |
|  |  | H9 | EI | EI | SLI | CLI | VLI | VLI | CLI | LI | EI |  |
