# Supplementary material for: Risk assessment based on a new decision-making approach with fermatean fuzzy sets
Source: PeerJ Comput Sci. 2025 Aug 28;11:e2990. doi: 10.7717/peerj-cs.2990 (PMC12453700; doi:10.7717/peerj-cs.2990)
Supplement: Supplemental Information 19 [file peerj-cs-11-2990-s019.docx]

| DM Weights | SDMG4 |  | H1 | H2 | H3 | H4 | H5 | H6 | H7 | H8 | H9 | CR |
| --- | --- | --- | --- | --- | --- | --- | --- | --- | --- | --- | --- | --- |
| 0.1817 | DM1 | H1 | EI | HI | SLI | SMI | LI | CLI | VLI | EI | EI | 0,079 |
|  |  | H2 | LI | EI | VLI | SLI | VLI | CLI | CLI | LI | EI |  |
|  |  | H3 | SLI | SMI | EI | HI | SLI | VLI | LI | SMI | VHI |  |
|  |  | H4 | SLI | SMI | LI | EI | VLI | CLI | CLI | SLI | EI |  |
|  |  | H5 | HI | VHI | SMI | VHI | EI | LI | SLI | HI | CHI |  |
|  |  | H6 | CHI | CHI | VHI | CHI | HI | EI | SMI | HI | CHI |  |
|  |  | H7 | VHI | CHI | HI | CHI | SMI | SLI | EI | HI | CHI |  |
|  |  | H8 | EI | HI | SLI | SMI | LI | LI | LI | EI | SMI |  |
|  |  | H9 | EI | EI | VLI | EI | CLI | CLI | CLI | SLI | EI |  |
| 0.1817 | DM2 | H1 | EI | HI | SLI | SMI | LI | CLI | VLI | HI | HI | 0,087 |
|  |  | H2 | LI | EI | VLI | SLI | CLI | CLI | CLI | EI | EI |  |
|  |  | H3 | SMI | VHI | EI | HI | SLI | VLI | LI | VHI | VHI |  |
|  |  | H4 | SLI | SMI | LI | EI | VLI | CLI | CLI | SMI | SMI |  |
|  |  | H5 | HI | CHI | SMI | VHI | EI | LI | SLI | CHI | CHI |  |
|  |  | H6 | CHI | CHI | VHI | CHI | HI | EI | SMI | CHI | CHI |  |
|  |  | H7 | VHI | CHI | HI | CHI | SMI | SLI | EI | CHI | CHI |  |
|  |  | H8 | LI | EI | SLI | SLI | CLI | CLI | CLI | EI | EI |  |
|  |  | H9 | LI | EI | VLI | SLI | SLI | SLI | SLI | EI | EI |  |
| 0.1817 | DM3 | H1 | EI | CLI | VLI | SLI | LI | VLI | SLI | SLI | SLI | 0,097 |
|  |  | H2 | CHI | EI | SMI | VHI | HI | HI | VHI | VHI | VHI |  |
|  |  | H3 | VHI | SLI | EI | VHI | HI | HI | VHI | VHI | VHI |  |
|  |  | H4 | SMI | VLI | VLI | EI | LI | LI | SLI | SLI | SLI |  |
|  |  | H5 | HI | LI | LI | HI | EI | EI | HI | HI | HI |  |
|  |  | H6 | VHI | LI | LI | HI | EI | EI | SMI | HI | VHI |  |
|  |  | H7 | SMI | VLI | VLI | SMI | LI | SLI | EI | SMI | SMI |  |
|  |  | H8 | SMI | VLI | VLI | SMI | LI | LI | SLI | EI | EI |  |
|  |  | H9 | SMI | VLI | VLI | SMI | LI | VLI | SLI | EI | EI |  |
| 0.1365 | DM5 | H1 | EI | SMI | LI | EI | SLI | CLI | VLI | EI | SMI | 0,059 |
|  |  | H2 | SLI | EI | CLI | SLI | LI | CLI | CLI | SLI | EI |  |
|  |  | H3 | HI | CHI | EI | HI | SMI | LI | SLI | HI | CHI |  |
|  |  | H4 | EI | SMI | LI | EI | SLI | CLI | VLI | EI | SMI |  |
|  |  | H5 | SMI | HI | SLI | SMI | EI | LI | SLI | SMI | CHI |  |
|  |  | H6 | CHI | CHI | HI | CHI | HI | EI | SMI | HI | CHI |  |
|  |  | H7 | VHI | CHI | SMI | VHI | SMI | SLI | EI | VHI | VHI |  |
|  |  | H8 | EI | SMI | LI | EI | SLI | LI | VLI | EI | SMI |  |
|  |  | H9 | SLI | EI | CLI | SLI | CLI | CLI | VLI | SLI | EI |  |
| 0.1365 | DM6 | H1 | EI | HI | CLI | EI | LI | CLI | VLI | SLI | SMI | 0,09 |
|  |  | H2 | LI | EI | CLI | LI | VLI | CLI | CLI | LI | SLI |  |
|  |  | H3 | CHI | CHI | EI | VHI | SMI | SLI | SLI | HI | CHI |  |
|  |  | H4 | EI | HI | VLI | EI | LI | CLI | VLI | SLI | SMI |  |
|  |  | H5 | HI | VHI | SLI | HI | EI | VLI | SLI | SMI | HI |  |
|  |  | H6 | CHI | CHI | SMI | CHI | VHI | EI | SMI | HI | CHI |  |
|  |  | H7 | VHI | CHI | SMI | VHI | SMI | SLI | EI | HI | CHI |  |
|  |  | H8 | SMI | HI | LI | SMI | SLI | LI | LI | EI | HI |  |
|  |  | H9 | SLI | SMI | CLI | SLI | LI | CLI | CLI | LI | EI |  |
| 0.1817 | DM7 | H1 | EI | SMI | SLI | EI | LI | VLI | LI | SLI | SMI | 0.042 |
|  |  | H2 | SLI | EI | VLI | SLI | VLI | CLI | VLI | LI | EI |  |
|  |  | H3 | SMI | VHI | EI | HI | SLI | LI | SLI | EI | VHI |  |
|  |  | H4 | EI | SMI | LI | EI | LI | VLI | LI | SLI | SMI |  |
|  |  | H5 | HI | VHI | SMI | HI | EI | SLI | EI | LI | CHI |  |
|  |  | H6 | VHI | CHI | HI | VHI | SMI | EI | SMI | HI | CHI |  |
|  |  | H7 | HI | VHI | SMI | HI | EI | SLI | EI | EI | VHI |  |
|  |  | H8 | SMI | HI | EI | SMI | HI | LI | EI | EI | VHI |  |
|  |  | H9 | SLI | EI | VLI | SLI | CLI | CLI | VLI | VLI | EI |  |
